# Analysis of completeness of COVID-19 notification forms among the Indigenous population in the State of Espírito Santo, 2020

**DOI:** 10.1590/S2237-96222025v34e20240176.en

**Published:** 2025-04-14

**Authors:** Priscila Carminati Siqueira, Carolina Maia Martins Sales, Thiago Nascimento do Prado, Ethel Leonor Noia Maciel

**Affiliations:** 1 Universidade Federal do Espírito Santo, Centro de Ciências da Saúde, Programa de Pós-Graduação em Saúde Coletiva. Vitória, ES, Brazil

**Keywords:** COVID-19, Indigenous Peoples, Epidemiology, Health Information Systems, Disease Notification, Covid-19, Pueblos Indígenas, Epidemiología, Sistemas de Información en Salud, Notificación de Enfermedades

## Abstract

**Objective:**

To evaluate the completeness of COVID-19 notification form data on the Indigenous population living in the state of Espírito Santo, Brazil, in 2020.

**Methods:**

This was a descriptive cross-sectional study carried out on COVID-19 notification data on the Indigenous population living in Espírito Santo in 2020. The scores used to assess completeness were: excellent (>95,0%), good (91,0%-95,0%), regular (81,0%-90,0%), poor (50,0%-80,0%) and very poor (<50,0%).

**Results:**

3,479 notification forms were analyzed. The sociodemographic variables, neighborhood and gender, and the symptom and comorbidity variables showed “excellent” completeness. The scores were “good” for disease classification and “regular” for ethnic group. The schooling variable, considered mandatory, was classified as “very poor”.

**Conclusion:**

The data analyzed had “excellent” completeness (65.3%). “Very poor” completeness was identified for 19.2% of the items evaluated, which shows that some items on the forms had a low standard of data recording.

## Resumen

**Objetivo:**

Evaluar la completitud de los datos de los formularios de notificación de Covid-19 de la población indígena residente en Espírito Santo, Brasil, en 2020.

**Métodos:**

Se trata de un estudio descriptivo transversal realizado con datos de notificación de Covid-19 en la población indígena viviendo en Espírito Santo en 2020. Las puntuaciones utilizadas para evaluar la integridad fueron: excelente (>95,0%), buena (91,0%-95,0%), regular (81,0%-90,0%), mala (50,0%-80,0%) y muy mala (<50,0%).

**Resultados:**

Se analizaron 3.479 notificaciones. Las variables sociodemográficas, barrio y género, y las variables de síntomas y comorbilidad mostraron una completitud “excelente”. Las puntuaciones fueron “buenas” para la clasificación de la enfermedad y “regulares” para la categoría étnica. La variable educación, considerada obligatoria, fue clasificada como “muy mala”.

**Conclusión:**

Los datos analizados presentaron una completitud “excelente” (65,3%). Se identificó una completitud “muy mala” en el 19,2% de los ítems evaluados, lo que demuestra que algunos ítems de los formularios presentaron un bajo estándar de registro de datos.

## Introduction

In 2020, there were 476 million Indigenous people worldwide, less than 6.0% of the global population (1). In Brazil, the original peoples totaled 1,693,535, being equivalent to 0.8% of the country’s total inhabitants (2). 

Brazil recorded 76,872 confirmed cases of COVID-19 among the Indigenous population in 2020; 969 of them died, with a case fatality ratio of 1.2% (3). Incidence and fatality indicators were higher in the Indigenous population when compared to non-Indigenous populations (4-6).

Indigenous people have great vulnerabilities and live with a high burden of chronic and infectious diseases, as well as deprivations (7-9). Infectious diseases in these groups spread quickly due to their collective lifestyle, poor sanitation conditions, and geographic location, which have contributed to increased COVID-19 complications and deaths (1,10-11).

In order to be able to understand the extent of the spread of COVID-19 in this population and its impact on their morbidity and mortality, a quality information system is essential. The objective of this study was to evaluate the completeness of COVID-19 notification form data on the Indigenous population living in Espírito Santo in 2020.

## Methods

### Design

This is a descriptive cross-sectional study of the completeness of COVID-19 notification forms regarding the indigenous population living in Espírito Santo in 2020.

### Background 

The state of Espírito Santo uses the official system for compulsory notification of diseases, health conditions and public health events by public and private health services, namely the Brazilian National Health System computerized health surveillance strategy, abbreviated as e-SUS VS (12).

**Figure 1:**
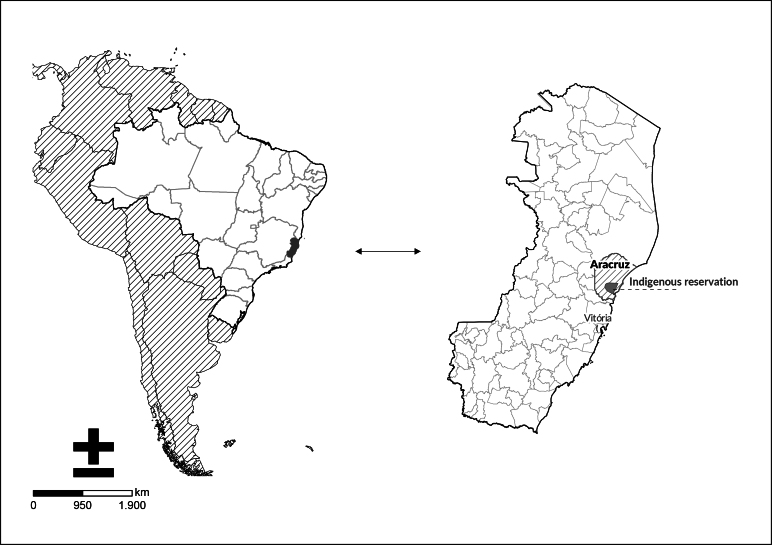
Indigenous territories. State of Espírito Santo, 2023

The state of Espírito Santo is located in the Southeast region of Brazil and has 78 municipalities. It forms part of the Minas Gerais/Espírito Santo Special Indigenous Health District. The Indigenous villages are located in Aracruz, in the north of the state, comprising 5,099 Indigenous people and 14 villages (Figure 1) (13-14).

### Participants

We investigated COVID-19 notifications relating to Indigenous villager dwellers.

### Variables

We considered the following variables for this study:

- Sociodemographic: neighborhood, age group, sex and schooling.
- Symptoms: fever, breathing difficulty, cough, runny nose, sore throat, diarrhea and headache.
- Comorbidities: pulmonary, cardiovascular, renal, diabetes, tobacco smoking and obesity.
- Disease confirmation and progression: classification, progression, confirmation criterion and case closure date.
- Other variables: hospitalization, journeys, health professional and person with a disability.
- Data sources and measurement: e-SUS VS database.

### Bias control

In order to minimize selection bias, we used all COVID-19 notifications for the Indigenous population living in Espírito Santo in 2020. Notifications with duplicate records were excluded.

### Study size

This study was based on 3,479 notifications.

### Statistic methods

The “data quality” item was used for the analysis (15-16). The scores used to categorize completion rates were: excellent (>95,0%), good (90,0%-95,0%), regular (80,0%-90,0%), poor (50,0%-80,0%) and very poor (<50,0%) (17-20).

For the final classification variable, categories left blank and the unspecified flu syndrome category were considered to be missing data categories. For the remaining variables, the blank and unknown categories (18-20) were considered to be missing data categories.

Descriptive statistics were performed using Stata (version 14). Absolute and relative frequency measures were calculated for the completeness categories of each item.

## Results

**Figure 2:**
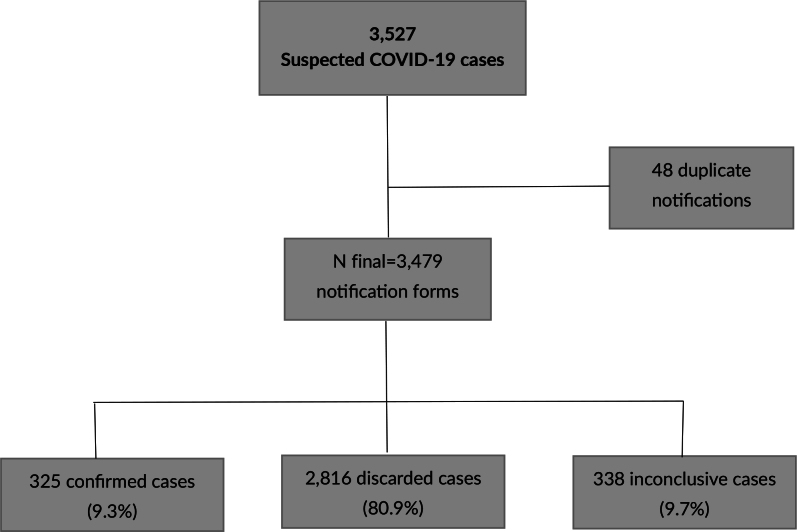
Notifications of suspected COVID-19 cases among Indigenous village dwellers. State of Espírito Santo, 2020

In 2020 in Espírito Santo, 3,527 suspected COVID-19 cases were reported for the Indigenous village population. Forty-eight of these notifications were duplicates, resulting in 3,479 records being analyzed. Confirmed cases of the disease accounted for 9.3%, and no deaths were recorded. Inconclusive cases accounted for 9.7%. Discarded cases accounted for 80.9% (Figure 2).

The epidemiological profile was comprised predominantly of women (51.6%), the Tupiniquim ethnic group (79.0%), and those between 21 and 40 years old (41.8%). The most prevalent symptoms were fever (35.8%), cough (30.1%), headache (30.1%) and sore throat (20.6%).

“Excellent” completeness was found for the sociodemographic variables neighborhood (99.9%) and gender (100.0%). The sociodemographic variable ethnic group stood out for its “regular” completeness (89.4%).

Regarding disease symptoms and comorbidities, all variables had “excellent” completeness (100.0%). Among the variables confirming COVID-19 and its progession, only the confirmation criterion variable had “excellent” completeness (95.6%). The classification and case closure date variables had a level of completeness considered “good”, with 90.2% and 90.4%, respectively. Variables with completeness greater than 95.0% accounted for 65.3% (17) of the total variables selected in the study (Table 1). 

**Table 1:** Absolute and relative frequency of completeness of COVID-19 notification forms for Indigenous village dwellers. State of Espírito Santo, 2020

Variables	n (%)	Classification
**Sociodemographic**		
Neighborhood	3,477 (99.9)	Excellent
Sex	3,479 (100,0)	Excellent
Ethnic group	3,112 (89.4)	Regular
Schooling	378 (10.8)	Very poor
Symptoms		
Fever	3,479 (100,0)	Excellent
Breathing difficulty	3,479 (100,0)	Excellent
Cough	3,479 (100,0)	Excellent
Runny nose	3,479 (100,0)	Excellent
Sore throat	3,479 (100,0)	Excellent
Diarrhea	3,479 (100,0)	Excellent
Headache	3,479 (100,0)	Excellent
Comorbidities		
Pulmonary	3,479 (100,0)	Excellent
Cardiovascular	3,479 (100,0)	Excellent
Renal	3,479 (100,0)	Excellent
Diabetes	3,479 (100,0)	Excellent
Tobacco smoking	3,479 (100,0)	Excellent
Obesity	3,479 (100,0)	Excellent
**Disease confirmation and progression**		
Classification	3,141 (90.2)	Good
Progression	260 (7.4)	Very poor
Confirmation criterion	3,328 (95.6)	Excellent
Case closure data	3,145 (90.4)	Good
**Other variables**		
Hospitalized	314 (9.0)	Very poor
Journey in Brazil	310 (8.9)	Very poor
International journey	314 (8.7)	Very poor
Health professional	2,826 (81.2)	Regular
Person with a disability	3,471 (99.7)	Excellent

There was no “poor” completeness among the variables examined in the study. The disease progression and schooling variables had a “very poor” level of completeness (7.8% and 10.8%). The other variables relating to hospitalization and to travel were also classified as “very poor” (Table 1)

## Discussion

This study analyzed notification data for the first year of the COVID-19 pandemic, which comprised the period with the highest incidence of the disease among Indigenous peoples in Espírito Santo (3). No deaths among them were recorded during this period. 

Espírito Santo has 14 Indigenous villages located in Aracruz, belonging to two ethnic groups: the Tupiniquins and the Guaranis (13-14). The layout of housing in Espírito Santo Indigenous villages differs from the traditional model. In the Tupi-Guarani villages of Espírito Santo, the houses are isolated from each other and connected by paths that spread through the forest (14). Isolation of homes may have contributed to the disease having spread less among the community.

When carrying out the completeness analysis, we found that the majority of data recorded on the e-SUS VS for COVID-19 among this population had an “excellent” rating (65.3%). “Very poor” completeness was identified in 19.2% of the items evaluated, showing that some items on the forms had a low standard of data recording.

The sociodemographic variables and those related to COVID-19 symptoms were mandatory, so that failure to fill out these fields made it impossible to complete the notification form (17,19,21), thus explaining the “excellent” completeness found in our analysis. Other regional studies that evaluated COVID-19 data completeness showed “excellent” completion of the symptoms, neighborhood and gender variables (18-19). Studies that analyzed the quality of notifications of other health conditions also demonstrated “excellent” completeness for the neighborhood and gender variables, corroborating the findings of this study (20,22).

In this study, the confirmation criterion and comorbidities variables stand out because their rating was “excellent” and because they are considered to be essential variables, so that filling them out is not mandatory (21). Another study carried out in Espírito Santo regarding COVID-19 among children and adolescents also found “excellent” completeness for comorbidity variables (19).

By identifying illnesses related to COVID-19, risk groups in Indigenous villages can be identified and prevention measures and disease control strategies can be adopted to reduce circulation of the virus. Groups with greater vulnerability (7,10,23) and high prevalence of chronic conditions are predisposed to greater susceptibility to coronavirus infection and greater fatality due to complications (4-6,24-25).

The sociodemographic variables ethnic group and schooling had “regular” and “very poor” completeness. These variables are mandatory (17-19,21). The lower completeness of these variables may result from filling out the field with the “unknown” option, resulting in low completeness. Several epidemiological studies of COVID-19 and other infectious diseases of public health importance in Brazil also found unsatisfactory completeness for the race/color and schooling variables (18-20,26-28). Defining the epidemiological and sociological profile is important for understanding the unequal and disproportionate impact of COVID-19 on the Indigenous population (29-30).

The disease progression variable had “very poor” completeness. Filling out this variable is extremely important for the outcome of the notification (21). Timely completion of this variable allows the case fatality ratio of the disease to be identified in the population, especially when it comes to a group at greater risk of becoming ill and dying from infectious diseases, such as the Indigenous population (4-9,29).

The case closure date variable had completeness classified as “good”. The information system has a limitation regarding the accuracy of the data entered for this variable, since closure of notifications occurred without the information necessary to finalize the cases in a properly recorded manner. The disease progression variable showed “very poor” completeness.

These gaps in notifications highlight failures in recording information and in monitoring cases by epidemiological surveillance teams. Such gaps show shortcomings in data feedback, which contributes to the incompleteness of the variables (18,22,28). The analyses presented in this article are important for verifying information system record status and data quality.

In order to improve the recording of COVID-19 notifications, the classification of case closure variables needs to be changed, reclassifying these fields as mandatory instead of essential. Periodic training and capacity building must also be provided for health professionals responsible for filling out notifications (18-20,26) and monitoring events in order to consolidate information held on the system. This contributes to the development of planning and resource management actions and to controlling the spread of the virus, especially in populations living in communities, with large families living together, which makes social isolation and access to health services difficult.
