# Supplementary material for: Analysis of completeness of COVID-19 notification forms among the Indigenous population in the State of Espírito Santo, 2020
Source: Epidemiol Serv Saude. 2025 Apr 14;34:e20240176. doi: 10.1590/S2237-96222025v34e20240176.en (PMC11998908; doi:10.1590/S2237-96222025v34e20240176.en)
Supplement: Translation

# Translation: Análise da completude das fichas de notificação de covid-19 na população indígena no Espírito Santo, 2020

**DOI:** 10.1590/S2237-96222025v34e20240176.pt

**Authors:** Priscila Carminati Siqueira, Carolina Maia Martins Sales, Thiago Nascimento do Prado, Ethel Leonor Noia Maciel

**Affiliations:** 1 Universidade Federal do Espírito Santo, Centro de Ciências da Saúde, Programa de Pós-Graduação em Saúde Coletiva, Vitória, ES, Brasil

**Keywords:** Covid-19, Povos Indígenas, Epidemiologia, Sistemas de Informação em Saúde, Notificação de Doenças

## Abstract

**Objetivo:**

Avaliar a completude dos dados das fichas de notificação de covid-19 da população indígena aldeada no Espírito Santo em 2020.

**Métodos:**

Tratou-se de estudo transversal descritivo realizado com dados de notificação de covid-19 na população indígena aldeada no Espírito Santo em 2020. Os escores utilizados para avaliar a completude foram: excelente (>95,0%), bom (91,0%-95,0%), regular (81,0%-90,0%), ruim (50,0%-80,0%) e muito ruim (<50,0%).

**Resultados:**

Foram analisadas 3.479 notificações. As variáveis sociodemográficas bairro e sexo e as variáveis de sintomas e de comorbidades apresentaram completude “excelente”. Os escores foram “bom” para a classificação da doença e “regular” para a categoria etnia. A variável escolaridade, considerada de preenchimento obrigatório, foi classificada como “muito ruim”.

**Conclusão:**

Os dados analisados apresentaram completude “excelente” (65,3%). Identificou-se completude “muito ruim” em 19,2% dos itens avaliados, o que evidencia que alguns itens nos formulários apresentaram baixo padrão de registro dos dados.

## Introdução

Em 2020, existiam 476 milhões de indígenas no mundo, menos de 6,0% da população mundial (1). No Brasil, os povos originários compreendiam 1.693.535 pessoas, o equivalente a 0,8% do total de habitantes (2). 

O Brasil registrou 76.872 casos confirmados de covid-19 na população indígena em 2020; destes 969 evoluíram a óbito, com a letalidade de 1,2% (3). Os indicadores de incidência e letalidades foram mais elevados na população indígena quando comparadas com as populações não indígenas (4-6).

Os indígenas apresentam grandes vulnerabilidades, convivem com elevada carga de doenças crônicas, infecciosas e carenciais (7-9). As doenças infecciosas nesses grupos se disseminam de forma rápida devido ao estilo de vida coletiva, condições precárias de saneamento, além da disposição geográfica, o que contribui para aumentar as complicações e mortes pela covid-19 (1,10-11).

Para compreender a extensão da disseminação da covid-19 nessa população e o impacto na morbimortalidade, é imprescindível apresentar um sistema de informação de qualidade. Este estudo teve por objetivo avaliar a completude dos dados das fichas de notificação da covid-19 da população indígena aldeada no Espírito Santo em 2020.

## Métodos

### Delineamento

Trata-se de estudo transversal descritivo da completude das notificações de covid-19 da população indígena aldeada no Espírito Santo em 2020.

### Contexto

No Espírito Santo, há o sistema oficial para notificação compulsória de doenças, agravos e eventos de saúde pública nos serviços públicos e privados no estado: a estratégia de informatização do Sistema Único de Saúde – vigilância em saúde (e-SUS VS) (12).

O estado está localizado na região Sudeste do Brasil. O Espírito Santo contém 78 municípios e pertence ao Distrito Sanitário Especial Indígena – Minas Gerais/Espírito Santo. Os indígenas aldeados estão localizados em Aracruz, no norte do estado, com 5.099 indígenas e 14 aldeias (Figura 1) (13-14).

**Figura 1:** Território indígena. Espírito Santo, 2023

### Participantes

Consideraram-se as notificações de covid-19 dos indígenas aldeados.

### Variáveis

As variáveis consideradas para este estudo foram as elencadas a seguir.

- Sociodemográficas: bairro, faixa etária, sexo e escolaridade.
- Sintomas: febre, dificuldade respiratória, tosse, coriza, dor de garganta, diarreia e cefaleia.
- Comorbidades: pulmonar, cardiovascular, renal, diabetes, tabagismo e obesidade.
- Confirmação da doença e evolução: classificação, evolução, critério de confirmação e data de encerramento.
- Outras variáveis: internação, deslocamento, profissional de saúde e deficiência.
- Fontes de dados e mensuração: banco de dados do e-SUS VS.

### Controle de viés

Para minimizar o viés de seleção, utilizaram-se todas as notificações de covid-19 na população indígena aldeada no Espírito Santo em 2020. Foram excluídas as notificações com duplicidade de registro.

### Tamanho do estudo

Utilizaram-se 3.479 notificações para o estudo.

### Métodos estatísticos

O item “qualidade dos dados” foi utilizado para a análise (15-16). Os escores utilizados para categorizar as taxas de completude foram: excelente (>95,0%), bom (90,0%-95,0%), regular (80,0%-90,0%), ruim (50,0%-80,0%) e muito ruim (<50,0%) (17-20).

Para a variável classificação final, consideraram-se como categorias de dados faltantes as categorias em branco e síndrome gripal não especificada. Para as demais variáveis, consideraram-se como categorias de dados faltantes as categorias em branco e ignorado (18-20).

A estatística descritiva foi realizada utilizando o programa Stata (versão 14). Medidas de frequência absoluta e relativa foram calculadas para as categorias de completude de cada item.

## Resultados

Em 2020 no Espírito Santo, foram notificados 3.527 casos suspeitos de covid-19 na população indígena aldeada. Quarenta e oito dessas notificações eram duplicadas, restando, assim, 3.479 fichas analisadas. Os casos confirmados para a doença representaram 9,3%, e não houve registro de óbito. Os casos inconclusivos corresponderam a 9,7%. Os casos descartados foram 80,9% (Figura 2).

**Figura 2:** Notificações dos casos suspeitos de covid-19 na população indígena aldeada. Espírito Santo, 2020

O perfil epidemiológico compreendeu mulheres (51,6%), da etnia tupiniquim (79,0%), entre 21 e 40 anos (41,8%). Os sintomas mais prevalentes foram febre (35,8%), tosse (30,1%), cefaleia (30,1%) e dor de garganta (20,6%).

Observou-se completude “excelente” para as variáveis sociodemográficas bairro (99,9%) e sexo (100,0%). A variável sociodemográfica etnia destacou-se com completude “regular” (89,4%). 

Em sintomas da doença e comorbidades, todos apresentaram completude “excelente” (100,0%). Entre as variáveis de confirmação da doença e evolução, somente a variável critério de confirmação apresentou completude “excelente” (95,6%). As variáveis classificação e data de encerramento apresentaram nível de completude considerado “bom”, com 90,2% e 90,4%. As variáveis com completude superior a 95,0% representaram 65,3% (17) do total das variáveis selecionadas no estudo (Tabela 1). 

**Tabela 1:** Frequências absoluta e relativa das completudes das fichas de notificação da covid-19 na população indígena aldeada. Espírito Santo, 2020

Variáveis	n (%)	Classificação
Sociodemográficas		
Bairro	3.477 (99,9)	Excelente
Sexo	3.479 (100,0)	Excelente
Etnia	3.112 (89,4)	Regular
Escolaridade	378 (10,8)	Muito ruim
Sintomas		
Febre	3.479 (100,0)	Excelente
Dificuldade respiratória	3.479 (100,0)	Excelente
Tosse	3.479 (100,0)	Excelente
Coriza	3.479 (100,0)	Excelente
Dor de garganta	3.479 (100,0)	Excelente
Diarreia	3.479 (100,0)	Excelente
Cefaleia	3.479 (100,0)	Excelente
Comorbidades		
Pulmonar	3.479 (100,0)	Excelente
Cardiovascular	3.479 (100,0)	Excelente
Renal	3.479 (100,0)	Excelente
Diabetes	3.479 (100,0)	Excelente
Tabagismo	3.479 (100,0)	Excelente
Obesidade	3.479 (100,0)	Excelente
**Confirmação da doença e evolução**		
Classificação	3.141 (90,2)	Bom
Evolução	260 (7,4)	Muito ruim
Critério de confirmação	3.328 (95,6)	Excelente
Data de encerramento	3.145 (90,4)	Bom
**Outras variáveis**		
Ficou internado	314 (9,0)	Muito ruim
Viagem no Brasil	310 (8,9)	Muito ruim
Viagem internacional	314 (8,7)	Muito ruim
Profissional saúde	2.826 (81,2)	Regular
Possui deficiência	3.471 (99,7)	Excelente

Não foi verificada completude “ruim” entre as variáveis no estudo. As variáveis evolução e escolaridade apresentaram nível de completude “muito ruim” (7,8% e 10,8%). As demais variáveis de internação e as referentes a deslocamentos também foram classificadas com completude “muito ruim” (Tabela 1).

## Discussão

Este estudo analisou os dados de notificações referentes ao primeiro ano da pandemia de covid-19, que compreendeu o período com a maior incidência da doença nos povos originários no Espírito Santo (3). Não houve registro de óbito nesse período. 

O Espírito Santo possui 14 aldeias indígenas localizadas em Aracruz, pertencentes a duas etnias: os tupiniquins e os guaranis (13-14). As disposições das moradias nas aldeias capixabas diferem do modelo tradicional. Nas aldeias tupi-guarani capixabas, as casas estão isoladas entre si e conectadas por caminhos que se espalham na mata (14). O isolamento das moradias pode ter contribuído para a menor disseminação da doença na comunidade.

Ao realizar a análise da completude, percebeu-se que a maioria dos dados registrados no e-SUS VS de covid-19 dessa população apresentou classificação “excelente” (65,3%). Identificou-se completude “muito ruim” em 19,2% dos itens avaliados, evidenciando que alguns itens nos formulários apresentaram baixo padrão de registro dos dados.

As variáveis sociodemográficas e as relacionadas com os sintomas da covid-19 foram de preenchimento obrigatório, logo a sua ausência impossibilitou a finalização da notificação (17,19,21), justificando a completude “excelente” na análise. Outros estudos regionais que avaliaram dados de completude da covid-19 apresentaram preenchimento “excelente” das variáveis sintomas, bairro e sexo (18-19). Estudos que analisaram a qualidade das notificações de outros agravos também demonstraram completude “excelente” para as variáveis bairro e sexo, corroborando os achados deste estudo (20,22).

Neste estudo, destacam-se as variáveis critério de confirmação e as de comorbidades por apresentarem classificação “excelente” e por serem variáveis essenciais e não de preenchimento obrigatório (21). Outro estudo realizado no estado referente à covid-19 em crianças e adolescentes também demonstrou completude “excelente” para as variáveis de comorbidades (19).

Ao identificar os agravos relacionados à covid-19, pode-se apontar os grupos de riscos nas aldeias e adotar medidas de prevenção e estratégias de controle da doença para diminuir a circulação viral. Os grupos de maior vulnerabilidade (7,10,23) e alta prevalência de condições crônicas predispõem à maior suscetibilidade à infecção pelo coronavírus e à maior letalidade por complicações (4-6,24-25).

As variáveis sociodemográficas etnia e escolaridade apresentaram completude “regular” e “muito ruim”. Essas variáveis são de preenchimento obrigatório (17-19,21). A menor completude dessas variáveis pode decorrer do preenchimento do campo com a opção “ignorado”, o que resultou na baixa completude. Diversos estudos epidemiológicos da covid-19 e de outras doenças infecciosas de importância para saúde pública no Brasil também apresentaram completude não satisfatória para as variáveis raça/cor e escolaridade (18-20,26-28). Definir o perfil epidemiológico e sociológico é importante para compreender o impacto desigual e desproporcional da covid-19 na população indígena (29-30).

A variável evolução apresentou completude “muito ruim”. O preenchimento dessa variável é de extrema importância para o desfecho da notificação (21). O preenchimento oportuno dessa variável permite identificar a letalidade da doença na população, principalmente quando se trata de um grupo com maior risco para adoecer e morrer por doenças infectocontagiosas, tal como a população indígena (4-9,29).

A variável data de encerramento apresentou completude classificada como “bom”. Verifica-se a limitação do sistema de informação quanto à precisão dos dados inseridos, uma vez que o encerramento das notificações ocorreu sem que as informações necessárias para a finalização dos casos estivessem devidamente registradas. A evolução da doença apresentou completude “muito ruim”.

Essas lacunas presentes nas notificações evidenciam falhas nos registros das informações e no acompanhamento dos casos pelas equipes de vigilância epidemiológica. Tais lacunas apresentam deficiência na retroalimentação dos dados, o que contribui para a incompletude das variáveis (18,22,28). As análises deste trabalho são importantes para verificar a situação dos registros e a qualidade dos dados existentes nos sistemas de informação.

Para melhorar o registro das notificações de covid-19, é necessário que ocorra a mudança na classificação das variáveis de encerramento, reclassificando esses campos considerados de essenciais para obrigatórios. Também deve ocorrer treinamentos e capacitações periódicas com os profissionais responsáveis pelo preenchimento das notificações (18-20,26) e monitoramento dos eventos para consolidar as informações no sistema. Isso contribui para o desenvolvimento de ações de planejamento e gestão dos recursos e para o controle da disseminação do vírus, principalmente nas populações que vivem em comunidades, em agregados familiares, o que dificulta o isolamento social e o acesso aos serviços de saúde.
